# Vitamin D in patients with intellectual and developmental disability in secure in-patient services in the North of England, UK

**DOI:** 10.1192/bjb.2017.8

**Published:** 2018-02

**Authors:** Iain McKinnon, Thomas Lewis, Naomi Mehta, Shahed Imrit, Julie Thorp, Chris Ince

**Affiliations:** 1 Secure Services, Northumberland Tyne and Wear NHS Foundation Trust, UK; 2 Institute of Neuroscience, Newcastle University, UK; 3 Older People’s Community Treatment Team, Northumberland Tyne and Wear NHS Foundation Trust, UK; 4 Learning Disability (CAMHS), Tees Esk and Wear Valleys NHS Foundation Trust, UK; 5 Autism Services, Northumberland Tyne and Wear NHS Foundation Trust, UK

## Abstract

**Aims and method:**

To assess the benefits of the introduction of routine vitamin D serum sampling for all patients admitted to a secure in-patient hospital in the North of England providing medium security, low security and rehabilitation services for offenders with intellectual and developmental disability. The vitamin D levels of 100 patients were analysed at baseline. Those with insufficient or deficient levels were offered treatment and retested after 1 year. Vitamin D levels were analysed in the context of level of security, seasonality of test and co-prescription of psychotropic medications.

**Results:**

Eighty-three per cent of patients had suboptimal vitamin D levels at initial test (41% deficient and 42% insufficient). This was seen among established patients and new admissions. Regression analysis of baseline vitamin D levels revealed no differences for levels of security, seasonality, whether patients were taking antipsychotic or anticonvulsant medication, or length of stay. Patients with deficiency or insufficiency were all offered supplementation. Those who opted in had significantly higher vitamin D levels at follow-up, compared with those who declined treatment.

**Clinical implications:**

Established and newly admitted patients in our secure mental health services had substantial levels of vitamin D insufficiency. In the light of the morbidities that are associated with deficient vitamin D levels, routine screening and the offer of supplementation is advisable.

**Declaration of interest:**

None.

## Background

Suboptimal levels of plasma 25-hydroxy vitamin D (25OHD) have been implicated in a number of morbidities, including musculoskeletal and cardiorespiratory diseases, diabetes and depression.^1^^,^^2^ A substantial proportion of vitamin D is obtained from ultraviolet-B light acting on the skin^1^, although the relative amounts obtained from solar and food sources vary from study to study.^3^ There is, therefore, a greater potential for impaired vitamin D levels during the autumn and winter months.^4^ Public Health England currently advise a minimum of 10 µg of vitamin D per day for the general population, but give no separate advice for those at risk of minimal sunlight exposure, such as institutionalised individuals.^5^

It is estimated that approximately 50% of the population of Northern England have insufficient or deficient vitamin D levels.^6^ Based on National Health Service guidelines, in January 2013, a decision was made by the clinicians at Northgate Hospital, Northumberland, UK, to routinely screen for vitamin D deficiency and treat as necessary.^7^ Northgate Hospital has a range of levels of secure care facilities for people with intellectual and developmental disabilities (IDD) who offend, as well as providing specialised services for people with severe autism spectrum disorders.

This paper describes an evaluation of the results of screening for 25OHD at Northgate Hospital. It was hypothesised that patients in secure services would have suboptimal levels of 25OHD due to less exposure to sunlight, and as a possible consequence of medications prescribed for mental disorders. We were also interested in the effect of season of testing, and whether the decision to routinely screen for and treat 25OHD deficiency would lead to improvement in any such deficiencies.

## Aims

1 To describe vitamin D levels in an ‘at risk’ population of offenders with IDD in secure care, across a range of levels of security;
2 To assess the effectiveness of screening for and treating any vitamin D deficiencies.

## Method

### Cohort 1

Between January 2013 and February 2014, all patients at Northgate Hospital were screened for vitamin D deficiency. These existing patients, who were having blood tests taken as part of their annual health checks over the year, had a serum vitamin D level added to the standard biochemistry request form with no additional venepuncture over and above routine testing.

### Cohort 2

While existing patients were having their vitamin D levels checked as part of annual health checks, new admissions to the secure services were also being screened. Because we judged that there may be differences between the established patient group and new admissions (higher levels of sun exposure, dietary intake differences), new admissions from January 2013 onwards were considered as a separate cohort for the purpose of assessing baseline vitamin D levels.

Data were collected for the first 100 patients screened, from both cohorts. The results were classified according to standard biochemical reference ranges for total 25OHD (the sum of 25OHD_2_ and 25OHD_3_):

• deficient (<25 nmol/L)
• insufficient (25–49 nmol/L)
• sufficient (50–75 nmol/L)
• optimal (>75 nmol/L) vitamin D levels.

Each result was stored with anonymised data about the patient's age, length of stay prior to the initial test, co-prescribed psychotropic medications, level of in-patient security and the season the test was performed.

Following the initial test, all patients were offered appropriate treatment by the hospital's physical health service if required, although not all patients accepted it. At Northgate Hospital, the supplementation protocol for vitamin D is based on guidance provided by the National Health Service Specialist Pharmacy Service.^8^ Those patients found to be deficient were treated with 20 000 units of oral colecalciferol twice weekly for 8 weeks and then maintained on 800 units daily. Those found to have insufficient levels were treated with a maintenance dose of 800–1600 units of oral colecalciferol daily. Patients' 25OHD levels were then re-tested a year later as part of the next year's annual health checks.

### Data analysis

#### Baseline 25OHD data

Mean baseline 25OHD levels were analysed using a single multiple regression analysis. The regression model assessed the effects of the following categorical predictors on baseline 25OHD levels: season of testing, levels of security, prescription of antipsychotic or anticonvulsant medication, and the cohort tested. Data that were not normally distributed were transformed appropriately (using a log_10_ transformation for 25OHD values).

### Effect of treatment

Patient records were scrutinised to ascertain whether, following baseline testing, treatment to correct any deficiency was offered and/or taken. Follow-up testing data were collected, and the McNemar test of marginal homogeneity was applied to the pairs of non-adjusted 25OHD levels to assess differences in patients’ 25OHD levels between baseline and follow-up.

Data were analysed using SPSS version 22.^9^ This service evaluation was registered with the Research and Development department of Northumberland, Tyne and Wear National Health Service Foundation Trust, in November 2013 (Registration number SER-13-018).

## Results

### Study population

Seventy-three established patents at Northgate Hospital had baseline 25OHD screening between January 2013 and February 2014 (cohort 1). Between January 2013 and July 2016, a further 27 patients had been admitted to the hospital and received baseline 25OHD screening on admission (cohort 2). The two cohorts are described in Tables 1 and 2. As expected, the median length of stay at baseline test was significantly greater in cohort 1 than in cohort 2 (36 months *vs.* 1 month; Mann–Whitney U: 34.5, *P* < 0.001) as new patients were tested soon after admission. There were no patients of Black and minority ethnic (BAME) origin among the patient group.

**Table 1:** Description of cohort 1

	*N*	Mean age	Sex		Antipsychotics *n* (%)	Anticonvulsants *n* (%)	Season of test (*n*)			
			M	F			Winter	Spring	Summer	Autumn
Medium security	20	26.5	20	0	9 (45)	8 (40)	5	6	6	3
Low security	39	38.2	30	9	15 (38)	10 (26)	9	7	16	7
Rehabilitation	9	34.0	8	1	4 (44)	2 (22)	0	1	5	3
Autism service	5	33.4	5	0	2 (40)	2 (40)	2	1	2	0
TOTAL	73	34.1			30	22	16	15	29	13

**Table 2:** Description of cohort 2

	*N*	Mean age	Sex		Antipsychotics *n* (%)	Anticonvulsants *n* (%)	Season of test (*n*)			
			M	F			Winter	Spring	Summer	Autumn
Medium security	14	25.8	14	0	3 (21)	2 (14)	2	4	6	2
Low security	11	35.7	9	2	4 (36)	5 (45)	4	1	2	4
Rehabilitation	1	50.0	0	1	1 (100)	0	0	1	0	0
Autism service	1	19.0	1	0	1 (100)	0	0	0	1	0
TOTAL	27	30.5			9	7	6	6	9	6

### Baseline vitamin D levels

**Fig. 1:**
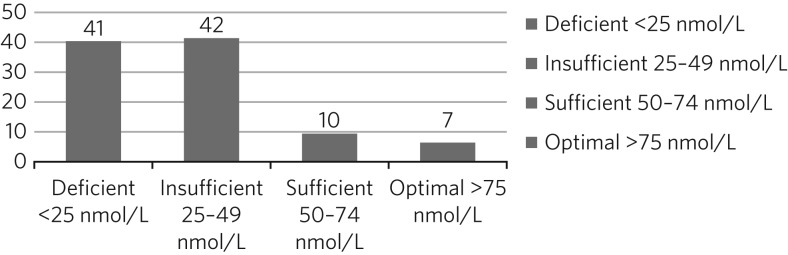
Vitamin D status of 100 patients at baseline testing.

The mean 25OHD level of the whole study population was 35.1 nmol/L (s.d. 28.1), and the median was 27.0 nmol/L, representing positively skewed values (Shapiro–Wilk: 0.699, *P* < 0.001). The regression analysis was therefore conducted using log_10_-transformed values. The statuses of 100 patients at baseline are represented in Fig 1, showing that the vast majority had either deficient or insufficient 25OHD levels.

The regression analysis demonstrated no effect of season or level of security on the vitamin D status of the patient at baseline Table 3). Furthermore, there were no differences between the two cohorts, or between those prescribed antipsychotic or anticonvulsant medication.

**Table 3:** Single multiple regression analysis of between-subject effects on log_10_ 25OHD levels at baseline

Factor	Log_10_ 25OHD type III sum of squares	d.f.	Mean square	F	*P*-value
Season	0.463	3	0.154	2.354	0.077
Security	0.147	3	0.049	0.746	0.527
Antipsychotics	0.171	1	0.171	2.600	0.110
Anticonvulsants	0.175	1	0.175	2.673	0.106
Cohort	0.089	1	0.089	1.350	0.248

There was a trend for lower 25OHD levels among patients already prescribed antipsychotic medication, and marginally higher levels in those on anticonvulsant medication, although four of the 29 patients in the latter group had already been prescribed vitamin D supplements. Furthermore, 25OHD levels were statistically non-significantly higher in the summer compared with winter (median 25OHD 33 nmol/L *vs.* 20 nmol/L), and in the rehabilitation service compared with the medium secure wards (median 25OHD 33.5 nmol/L *vs.* 27.5 nmol/L).

### Follow-up and treatment effects

**Fig. 2:**
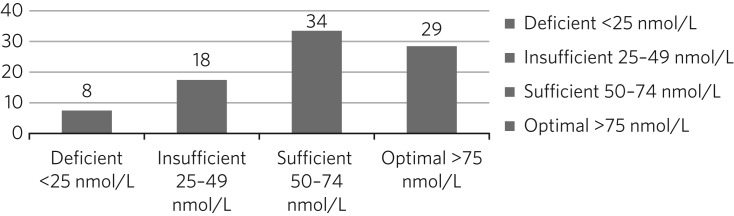
Vitamin D status of 89 patients at follow-up.

Follow-up data were available for 89 patients who were retested around 12 months after baseline screening. The mean 25OHD level was 66.3 nmol/L (s.d. 33.6) and the median was 62.0 nmol/L, which represented a less extreme but still significant positive skew (Shapiro-Wilk: 0.914, *P* = 0.035). The numbers of patients with sufficient and optimal 25OHD had improved substantially (see Fig 2).

**Fig. 3:**
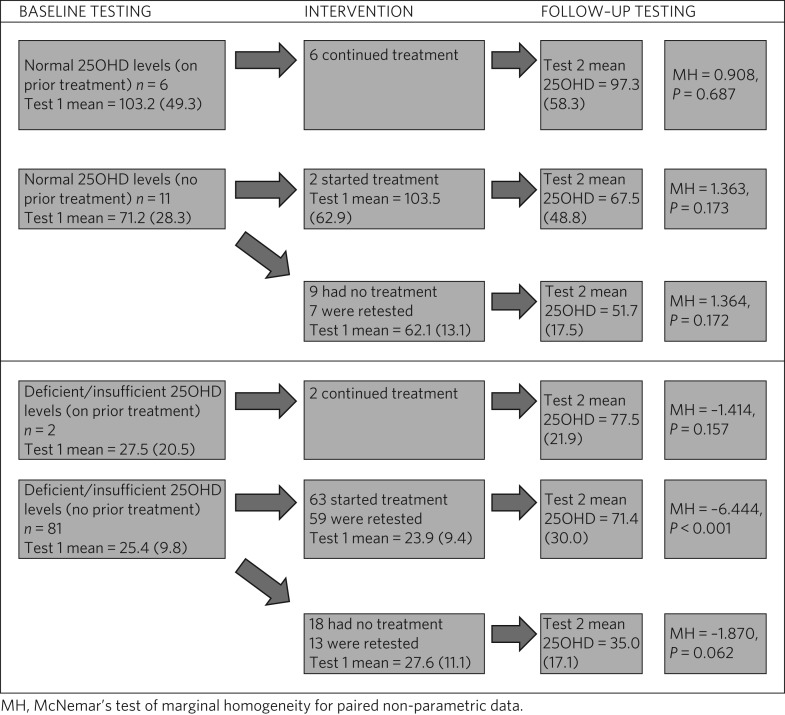
Flowchart of 100 patients who underwent baseline screening. Mean (s.d.) 25OHD levels are reported.

Not all patients who received baseline 25OHD screening and/or treatment were tested a second time. Some patients were discharged from hospital in the interim, or their 25OHD level was not requested by the physical treatment service. A flowchart of all 100 patients is provided in Fig 3.

The regression analysis was repeated for log_10_-transformed 25OHD levels at follow-up testing. As in the baseline data, there were no effects for seasonality, security level, or the prescription of antipsychotic or anticonvulsant medication.

## Discussion

This is the first study to report data from a specialist hospital for patients with IDD who offend, and those with severe autism spectrum disorders, with a number of levels of secure and non-secure wards and units. This paper describes routinely collected data on serum 25OHD levels obtained at baseline prior to clinical decisions to treat any vitamin D deficiency, along with follow-up serum levels after a year. The rationale for the implementation of this protocol was based on concerns that low levels of sun exposure, the secure environment and the prescription of psychotropic medication put in-patients in a ‘high risk’ category, with potentially serious long-term health sequelae.^7^

The data suggest considerable vitamin deficiency among this patient group. Furthermore, there were no clear differences in 25OHD levels between patients already in hospital when baseline screening took place and newly admitted patients. This might be attributed to deficiencies in the community, but, in a tertiary service, new admissions frequently transfer from other in-patient services. Limitations in the data available mean that further scrutiny is outside the scope of this paper. There appeared to be a trend for marginally lower 25OHD levels in secure wards compared with rehabilitation services, but there was no statistically significant variation.

Although no significant differences were found between patients taking psychotropic medication and those who were not, these factors remain of clinical concern with respect to bone health. A recent small-scale prospective study found that antipsychotics may inhibit vitamin D-metabolising enzymes, thereby causing a reduction in both calcium and 25OHD levels.^10^ This association is of particular concern given the known link between antipsychotics and osteoporosis risk via raised prolactin levels.^11^

Patients with normal 25OHD levels at baseline who were already on supplements all opted to continue with treatment. Those with normal levels at baseline who were not already receiving treatment were advised by their general practitioner based on the clinical scenario. Neither the supplemented nor the non-supplemented group had significant changes in their 25OHD status at follow-up, but numbers here were small.

Patients with suboptimal 25OHD levels were all offered supplementation using a standard protocol. Those who opted for supplementation had significantly higher 25OHD levels at follow-up, whereas those who opted out experienced non-significant changes. As the data here reported are routinely collected, it is not possible to make any systematic inference as to what lay behind a patient's decision to accept or decline supplementation.

Although vitamin D deficiency was widespread among this group, it was not present among all patients. There is some evidence from genome-wide association studies that genetic factors also have a significant role in identifying those at increased risk, but this is beyond the scope of this evaluation.^12^

Our data are commensurate with a study of psychiatric in-patients in a Scottish high-secure hospital at a similar geographical latitude to Northgate Hospital. That study concluded that all patients in secure settings should be screened and offered supplementation based on ‘significant and serious’ deficiency of 25OHD associated with bone demineralisation.^13^ Furthermore, such deficiencies do not appear to be limited to secure care. One study of people with IDD in institutionalised nursing care in Finland demonstrated significant 25OHD insufficiency, which was addressed by oral or intramuscular supplementation.^14^

The data in this study were routinely collected and not prospectively planned, leading to limitations in their interpretation. Serum parathyroid hormone and calcium levels were not routinely collected, nor were any bone mineralisation tests performed. No data on the content or vitamin D qualities of patients’ diets were collected. It is also not possible to comment on the longer-term health risks such as fractures without a larger, more specialised study. The patient group at Northgate is not ethnically diverse; all patients in this cohort were of white ethnicity. One study of a long-stay psychiatric in-patient facility found an association between low vitamin D levels and being of a BAME background, with improvements in 25OHD levels after treatment.^15^ Another recent study in Tier 4 adolescent psychiatric services showed similar numbers of white and BAME patients with 25OHD deficiency (46 vs. 53%).^16^

This raises the question as to whether all patients who are ‘high risk’ should be offered treatment. The long stays of patients within secure services, along with concomitant medication, would put these patients within this category. Current guidance by Public Health England recommends a minimum of 10 µg per day for the general population aged four and above, where diet alone is insufficient.^5^ However, the choice between simple supplementation or a deficiency protocol for patients such as these has not been fully established. Furthermore, it has not been established whether supplementation should be offered without suitable baseline screening. It should also be considered that the more general effects of vitamin D supplementation are far from certain. A meta-analysis of 18 randomised controlled trials (RCTs) suggested that there was a modest reduction in overall mortality for people taking standard doses of 25OHD supplementation,^17^ but a more recent umbrella review of systematic reviews and meta-analyses of observational studies and RCTs found little convincing evidence of a clear role for vitamin D in health outcomes.^18^

Nevertheless, this study has shown that in-patients with IDD appear to have deficiencies in vitamin D and that these are amenable to correction by oral supplementation in many cases. We recommend further research in this area, including prospective studies of the longer-term health sequelae.

## About the authors

**Iain McKinnon**, Consultant Psychiatrist, Northumberland, Tyne and Wear NHS Foundation Trust, and Honorary Senior Lecturer in Psychiatry, Institute of Neuroscience, Newcastle University, UK; **Thomas Lewis**, Specialist Registrar in Psychiatry, Northumberland, Tyne and Wear NHS Foundation Trust, UK; **Naomi Mehta**, Consultant Psychiatrist, Tees, Esk and Wear Valleys NHS Foundation Trust, UK; **Shahed Imrit**, Specialty Doctor, Northumberland, Tyne and Wear NHS Foundation Trust, UK; **Julie Thorp**, Consultant Psychiatrist, Northumberland, Tyne and Wear NHS Foundation Trust, UK; **Chris Ince**, Consultant Psychiatrist, Specialist Services, Northumberland, Tyne and Wear NHS Foundation Trust, UK.
